# Supplementary material for: Home-based nurturing care practices for children under five with low socioeconomic position in Santo Domingo, Dominican Republic
Source: PLoS One. 2025 Feb 11;20(2):e0314432. doi: 10.1371/journal.pone.0314432 (PMC11813109; doi:10.1371/journal.pone.0314432)
Supplement: S1 File — (PDF) [file pone.0314432.s001.pdf]

# MODIFIED WORKING MODEL OF THE CHILD INTERVIEW (2016)

Charles H. Zeanah, M.D.
Angela Breidenstine, Ph.D.
Kathryn L. Humphreys, Ph.D.
Richelle Long, Ph.D.

Section of Child & Adolescent Psychiatry
Tulane University School of Medicine
New Orleans, Louisiana, USA

Correspondence: Charles H. Zeanah, M.D.
Department of Psychiatry & Behavioral Sciences
Section of Child & Adolescent Psychiatry
Tulane University School of Medicine
1430 Tulane Avenue #8055
New Orleans, Louisiana 70112-2715
Ph.: (504) 988-5402
FAX: (504) 988-4264
email: czeanah@tulane.edu

The Modified Working Model of the Child Interview is a semi-structured interview to assess parents' internal representations or working models of their relationship to a particular child. The setting of the interview should be comfortable enough to allow for attention to the questions posed and a relaxed atmosphere that permits the opportunity for reflection.

The Modified WMCI (M-WMCI) includes several sections from the WMCI. We are evaluating its usefulness and validity at present and consider it to be under development. Use in research with permission of the first author only.

# MODIFIED WORKING MODEL OF THE CHILD INTERVIEW

**We are interested in how parents think and feel about their young children. This interview is a way for us to ask you about [CHILD] and your relationship with him/her.**

1. **I'd like you to describe [CHILD]'s personality now.** (Give the parent/caregiver enough time to respond to this before proceeding to specific descriptors below).
   a. **Pick five words (or phrases) to describe [CHILD]'s personality. After you have told me the five words, I will ask you to think about a specific memory that shows why you chose each of them.** (You may tell the parent/caregiver that it is fine to use any of the descriptors they used in response to the general probe above, but do not remind them about what those descriptors were. Phrases instead of words are fine. It is important not to rephrase what the interviewee says, but to use the words or phrases they stated. Some people have a hard time coming up with five words. Give them a "few more minutes" to think. If you feel they cannot come up with five, then move on. The numbers are less important than the descriptors. It does not matter if the words are synonyms. If the person cannot give five, you can say: "That's fine, if you think of another, we can come back to it.")
      i.
      ii.
      iii.
      iv.
      v.

      **Now I want you to try to remember one particular moment or memory that shows why you chose each of these words to describe [CHILD]'s personality.**
   b. For each word:
      i. **The first word you chose was [adjective]. Tell me one specific memory to show me why you chose [adjective] to describe [CHILD]'s personality**.
         The goal is to guide parent/caregivers to think of specific memories of their child. If the parent/caregiver struggles to identify a specific incident, especially on the first few words, follow up with:
         1. **What I would really like for you to do is tell me *one specific memory* that stands out to you that shows that [CHILD] is [Adjective].**

2. **Tell me about the last time [CHILD] did something that made you think he/she is [adjective].** (If you have asked all of the prompts, say: **I am going to be asking you for a *specific* memory for each of the words you have chosen.**)

2. a. **At this point, whom does [CHILD] remind you of?**
   - i. **In what ways?**
   - ii. **When did you first notice the similarity?**

   The following questions should be asked whether or not the parents have been mentioned.
   - iii. **Which of his/her parents is [CHILD] most like now?**
   - iv. **In what ways is [CHILD]'s personality like your personality?**
   - v. **In what ways is [CHILD]'s personality like his/her other parent's personality?**
   - vi. **Are there any family characteristics on your side that you see in [CHILD]'s personality?**
   - vii. **Are there any family characteristics on the other parent's side that you see in [CHILD]'s personality?**
3. **Name**
   - a. **How did you decide on [CHILD]'s name?**
   - b. **How well does the name seem to fit?**

4. **What do you feel is unique, different, or special about [CHILD]?** (Give ample opportunities for the interview to respond—this is a question to linger on rather than move through quickly. So, if there is a very brief response (or no response) consider saying things like, "That's okay, take your time," or "Do you want to add anything else?" or "Anything you want to add?"). If needed: **Do you notice anything that is different from other children?)**

5. **What about [CHILD]'s behavior is the most difficult for you to handle?** Pause. **Can you give an example of when he/she does this?** (If they deny challenges, follow up with "**Most parents find some things a little challenging…**")
   - a. **How often does this occur?**
     - i. **What do you do when [CHILD] acts that way?**
     - ii. **What do you *want to do* when [CHILD] [does the behavior]?**
     - iii. **How does it make you feel (on the inside) when [CHILD] [does the behavior]?**
   - b. **Does [CHILD] know you don't like it when he/she [does the behavior]?**

i. **Why do you think he/she does it?**

c. **What do you imagine will happen to [the behavior] as your child grows older?**

i. **Why do you think so?**

6. a. **Now I want you to describe your relationship with [CHILD].** (Give the parent/caregiver enough time to respond to this before proceeding to specific descriptors below).

a. **As before, I am going to again ask you to pick five words (or phrases), but this time to describe your relationship with [CHILD]. After you have told me the five words, I will ask you to think about a specific memory that shows why you chose each of them.**

i.
ii.
iii.
iv.
v.

**Now I want you to think of one particular moment or specific memory that shows why you chose each of these words to describe your relationship.**

b. For each word:

i. **The first word you chose was [adjective]. Tell me *one specific memory* that shows why you chose [adjective]**.

The goal here is again to guide parent/caregivers to think of specific memories of their child. If the parent/caregiver struggles to identify a specific incident, especially on the first few words, follow up with:

1. **What I would really like for you to do is tell me *one specific memory* that stands out to you that shows why you chose [adjective] to describe your relationship**.
2. **Tell me about the last time your relationship was [adjective].** (If you have asked all of the prompts, say: **I am going to be asking you for a *specific* memory for each of the words you have chosen.**)

7. **What do you enjoy most about your relationship with [CHILD]?** (Probe for specifics. Some general probes that you can ask include, "**Tell me more**." "**How so?**")

a. **What do you wish you could change about it?** (Again, probe for specifics. If interviewee says, "Nothing," ask "**Is there anything that you wish were a little bit different?"** Only probe once here)
b. **How do you feel your relationship with [CHILD] has shaped or affected his/her personality? (**Be sure to give ample time to respond to this question. If the parent/caregiver says "**Nothing**," probe [only] once "**Are you sure**?")
c. **Has your relationship with [CHILD] changed at all over time?** (If the parent/caregiver says "No," you can follow up with, "**Not at all**?" If the parent does not think the relationship has changed at all, move on to question 7)
    i. If yes: **In what ways?**
    ii. **How do you feel about the change in your relationship?**

8. **Which parent or caregiver is [CHILD] closest to now?**
    a. **How can you tell?**
    b. **Has it always been that way?**
    c. **Do you expect that the person [CHILD] is closest to will change (as he/she gets older, for instance)?**
        i. If yes: **How do you expect it to change?**

9. **Tell a favorite story about [CHILD], perhaps one you've told to family or friends.** (If the parent/caregiver is struggling, you may say: **I'll give you a minute to think about this one.** or **This doesn't have to be *the* favorite story, just *a* favorite story**.)
    a. **What do you like about this story?**

10. **I'd like you to think for a moment of [CHILD] as an adult.**
    a. **What fears do you have for his/her future?**
    b. **What do you hope for in his/her future?**
