# Supplementary material for: Home-based nurturing care practices for children under five with low socioeconomic position in Santo Domingo, Dominican Republic
Source: PLoS One. 2025 Feb 11;20(2):e0314432. doi: 10.1371/journal.pone.0314432 (PMC11813109; doi:10.1371/journal.pone.0314432)
Supplement: Attachment — Submitted filename: Response to Reviewers 19JULY2024.docx [file pone.0314432.s004.docx]

PONE-D-23-41977

Response to reviewers

1. Please ensure that your manuscript meets PLOS ONE's style requirements, including those for file naming. The PLOS ONE style templates can be found at

<https://journals.plos.org/plosone/s/file?id=wjVg/PLOSOne_formatting_sample_main_body.pdf> and

<https://journals.plos.org/plosone/s/file?id=ba62/PLOSOne_formatting_sample_title_authors_affiliations.pdf>

2. Please include a complete copy of PLOS’ questionnaire on inclusivity in global research in your revised manuscript. Our policy for research in this area aims to improve transparency in the reporting of research performed outside of researchers’ own country or community. The policy applies to researchers who have travelled to a different country to conduct research, research with Indigenous populations or their lands, and research on cultural artefacts. The questionnaire can also be requested at the journal’s discretion for any other submissions, even if these conditions are not met. Please find more information on the policy and a link to download a blank copy of the questionnaire here: <https://journals.plos.org/plosone/s/best-practices-in-research-reporting>. Please upload a completed version of your questionnaire as Supporting Information when you resubmit your manuscript.

3. In the online submission form, you indicated that [Data cannot be shared publicly because of they are identifiable qualitative data. Data are available upon request from the corresponding author for researchers who meet the criteria for access to confidential data.].

All PLOS journals now require all data underlying the findings described in their manuscript to be freely available to other researchers, either 1. In a public repository, 2. Within the manuscript itself, or 3. Uploaded as supplementary information.

This policy applies to all data except where public deposition would breach compliance with the protocol approved by your research ethics board. If your data cannot be made publicly available for ethical or legal reasons (e.g., public availability would compromise patient privacy), please explain your reasons on resubmission and your exemption request will be escalated for approval.

*Following the ethics committee protocol approved by Tulane University (2019-2375), the audio files and transcripts, which contain sensitive information and are provided in conditions of confidentiality, cannot be shared. This follows the recommendations made by Martijn de Koning and colleagues in "Guidelines for anthropological research: Data*

*management, ethics, and integrity" (Ethnography, 2019. 20(2): p. 170-174).*

*I can provide de-identified qualitative data coded and converted to numerical form.*

4. Please include captions for your Supporting Information files at the end of your manuscript, and update any in-text citations to match accordingly. Please see our Supporting Information guidelines for more information: <http://journals.plos.org/plosone/s/supporting-information>.

5. Please review your reference list to ensure that it is complete and correct. If you have cited papers that have been retracted, please include the rationale for doing so in the manuscript text, or remove these references and replace them with relevant current references. Any changes to the reference list should be mentioned in the rebuttal letter that accompanies your revised manuscript. If you need to cite a retracted article, indicate the article’s retracted status in the References list and also include a citation and full reference for the retraction notice.

Response to Reviewers' Comments:

1. Is the manuscript technically sound, and do the data support the conclusions?

The manuscript must describe a technically sound piece of scientific research with data that supports the conclusions. Experiments must have been conducted rigorously, with appropriate controls, replication, and sample sizes. The conclusions must be drawn appropriately based on the data presented.

Reviewer #1: Yes

Reviewer #2: Yes

Reviewer #3: Yes

2. Has the statistical analysis been performed appropriately and rigorously?

Reviewer #1: Yes

Reviewer #2: N/A

Reviewer #3: I Don't Know

3. Have the authors made all data underlying the findings in their manuscript fully available?

The [PLOS Data policy](http://www.plosone.org/static/policies.action#sharing) requires authors to make all data underlying the findings described in their manuscript fully available without restriction, with rare exception (please refer to the Data Availability Statement in the manuscript PDF file). The data should be provided as part of the manuscript or its supporting information, or deposited to a public repository. For example, in addition to summary statistics, the data points behind means, medians and variance measures should be available. If there are restrictions on publicly sharing data—e.g. participant privacy or use of data from a third party—those must be specified.

Reviewer #1: No

Reviewer #2: Yes

Reviewer #3: No

4. Is the manuscript presented in an intelligible fashion and written in standard English?

PLOS ONE does not copyedit accepted manuscripts, so the language in submitted articles must be clear, correct, and unambiguous. Any typographical or grammatical errors should be corrected at revision, so please note any specific errors here.

Reviewer #1: Yes

Reviewer #2: Yes

Reviewer #3: Yes

Review Comments to the Author

**Reviewer #1:** A very well versed and detailed article and research conducted in a developing country. Nurturing framework is very pivotal and much needed globally such kind of studies support the widespread implementation of ECD and NCF. The study uniquely highlighted intentional learning and corporal punishment. However, following are the points of feedback:

The title needs to be modified as research question and study is about perceptions of mothers in DR on NCF, weather they provide such care or not however title suggest home based NCF as an intervention in homes).

*Thank you for this observation. We have changed the title to: Home-based nurturing care practices for children under five with low socioeconomic position in Santo Domingo, Dominican Republic*

Introduction (1.1, 1.2, 1.3 can be combine into one short paragraph).

*We have combined these subsections into one sub-section entitled “The Nurturing Care Framework”, however, we did not make it into one paragraph as that would be very long.*

1.6 (Add DR country profile overall, what is the status of ECD strategies, why this country was selected for study, why there is high prevalence 14.5%, what is the current situation of government interventions?)

*Thank you for this comment. We have added in a sentence about why the DR was chosen for this study. There are few studies on neurodevelopment in the DR and so we cannot point to evidence as to why the prevalence of delay is high beyond the reasons we have offered (high rates of poverty, low levels of early learning opportunities). The second paragraph of section 1.6 (now 1.4) describes available government interventions. We do not have access to evaluation data on INAIPI but hope that will become available within the next few years.*

Discussion: Adding other developing countries data literature to support the study.

*Thank you for this comment. We agree and have added in findings from other low-income countries about responsive caregiving education, physical punishment, and the use of screens and well as information from systematic reviews and metanalyses.*

Conclusion: Adding recommendations as to government role and policies, input of WHO and other NGOs.

*Thank you, we have added in some language about strengthening institutions and WHO recommendations into the conclusion.*

**Reviewer #2**: The author cannot explain how the empirical findings contribute to the broader literature; discussing on how the findings address a gap in the literature, extend our current knowledge, or provide new insights into a specific phenomenon or problem.

*The authors thank you for this comment. We have included additional literature on the primary topic areas we contribute to the knowledge in the field in the discussion section. We have also included a summary paragraph in the conclusion section highlighting key take-aways. Our findings suggest caregivers are not empowered to teach their children and therefore seek distal tools for entertainment and education. This may curtail with our findings that young and adolescent mothers in this setting are using physical punishment because of their own feelings of helplessness and insecurity in their motherhood skills. These findings provide a clear path forward to build confidence and knowledge among young mothers. Additionally, we provide evidence that ethnographic research about caregiving practices may facilitate promoting nurturing care from local experiences.*

**Reviewer #3**: Congratulation on your work and the writing. My comment is submitted on the PLOS ONE platform. Please refer to the comments and please revise it before you resubmit the manuscript, especially using the right term in scientific writing. All the best and good luck.

*I have been unable to view these suggested revisions and we’re looking forward to incorporating them.*
