# Supplementary material for: Home-based nurturing care practices for children under five with low socioeconomic position in Santo Domingo, Dominican Republic
Source: PLoS One. 2025 Feb 11;20(2):e0314432. doi: 10.1371/journal.pone.0314432 (PMC11813109; doi:10.1371/journal.pone.0314432)
Supplement: Attachment — Submitted filename: Response to Reviewers_2.docx [file pone.0314432.s005.docx]

Response to Review 2

11/7/24

- It appears that 'Table 2. Select excerpts' could be better organised for clarity.

*Thank you for noticing this. The subthemes are in the same order they are presented in the paper. However, we re-read the citations and removed/ moved some to make sure each was consistent with its theme. We also added a short summary of the theme for each.*

- It is better to include protocol numbers in the ethics approval statement (i.e., 'We received approval from the Universidad Iberoamericana Human Research Protection Program and the Tulane University Research Ethics Committee').

*Yes, we have added the protocol numbers into this version.*

- Although detailed quotes provide context, some are lengthy, particularly in lines 596-611 and 636-655. Given the abundance of quotes in Table 2, consider condensing them to improve readability and avoid excess detail.

*We have condensed these citations as suggested.*

- In your submission, there are two clean copies and one trach change. Could you please do only one clean copy and one-track change?

*Yes, please forgive this oversight. We have corrected this in the current submission.*

Also, a note to say I have recently accepted a position at Yale University and they have a open source agreement with PLOS ONE, whereas Tulane has recently suspended theirs. Is it possible to change my affiliation from Tulane to Yale for this purpose?
